# Biofortification (Se): Does it increase the content of phenolic compounds in virgin olive oil (VOO)?

**DOI:** 10.1371/journal.pone.0176580

**Published:** 2017-04-27

**Authors:** Roberto D’Amato, Primo Proietti, Andrea Onofri, Luca Regni, Sonia Esposto, Maurizio Servili, Daniela Businelli, Roberto Selvaggini

**Affiliations:** Department of Agricultural, Food and Environmental Sciences—DSA3, University of Perugia, Perugia, Italy; SERGAS and IDIS, SPAIN

## Abstract

Extra-Virgin Olive Oil (EVOO) is a fundamental component of the Mediterranean diet and it may contain several anti-oxidant substances, such as phenols. Previous research has shown that this food may be enriched in phenols by spraying a sodium-selenate solution (100 mg L^-1^ Se) onto the crop canopy before flowering. The aim of this research was to evaluate the effect of this Se-fertilization before flowering (cv. Leccino) on the phenolic profile of EVOOs, and test to what extent such effects depend on the weather pattern, as observed in two contrasting experimental seasons (2013 and 2014). Results showed that Se-fertilisation enriched EVOOs both in selenium (up to 120 μg kg^-1^) and in phenols (up to 401 mg kg^-1^). This latter enrichment was related to an increase in PAL (L-Phenylalanine Ammonia-Lyase) activities and it was largely independent on the climatic pattern. Considering the phenolic profile, oleacein, ligustroside, aglycone and oleocanthal were the most affected compounds and were increased by 57, 50 and 32%, respectively. All these compounds, especially oleacein, have been shown to exert a relevant anti-oxidant activity, contributing both to the shelf-life of EVOOs and to positive effects on human health. It is suggested that Se-fertilisation of olive trees before flowering may be an interesting practice, particularly with poor cultivars and cold and rainy weather patterns, which would normally lead to the production of EVOOs with unfavourable phenolic profile.

## Introduction

In recent years, innovation in food industry has mainly been related to new scientific and technical approaches to food processing and to the introduction of novel 'functional' foods. The relevance of this latter type of food relates to the increasing cost of healthcare, increase in life expectancy, and the desire of elderly people for an improved quality of life in their latest years [1].

Most early developments in the above respect are related to food fortified with vitamins and/or minerals such as vitamin C, vitamin E, folic acid, zinc, iron, and calcium [2]. Subsequently, the focus shifted to food fortified with various micronutrients such as omega-3 fatty acids, phytosterols, and soluble fibre to promote good health or to prevent cancer and other dangerous diseases [3]. More recently, food companies have taken further steps to develop food offering multiple health benefits in a single product [4].

Within the above frame, olive oil plays an important role in human diet and health, in the whole Mediterranean Area, thanks to its high contents of monounsaturated fatty acids and functional compounds, including tocopherols, carotenoids, phospholipids, and phenols. In this manuscript we will focus on these latter compounds, which have shown very good antioxidant properties and thus may help protect the organism from reactive species [5] and reduce the risk of atherosclerosis and cardiovascular diseases [6; 7]. In addition, phenolic compounds can have beneficial effects also on the sensorial characteristics of olive oil, by protecting it against oxidation [8]. There is a strong interest in producing virgin olive oil (VOO) or extra-virgin olive oil (EVOO) with high content in phenolic compounds, in order to increase the human daily assumption of these beneficial substances [9].

Phenolic compounds, including lignin, flavonoids, phytoalexins, tannins, originate from the metabolism of phenylpropanoids (PPs), which belong to the largest group of secondary metabolites produced by plants, also in response to biotic and abiotic stresses [10; 11]. L-Phenylalanine Ammonia-Lyase (PAL, EC 4.3.1.5) is the key enzyme for this biosynthetic pathway and its activity shows considerable variation according to the development stage of the olive fruit [12], as well as to possible stresses of various nature, such as irradiation, wounding, nutrient deficiencies, water shortage, herbicide treatment and viral, fungal, and insect attacks [13]. In general, the accumulation of phenolic compounds in olive drupes is a highly variable process, depending on the physiological state of the plant/fruit and it is the result of a complex balance between phenol biosynthesis and catabolism [14]. Furthermore, it should also be taken into account that, despite a wide range of phenolic compounds have been identified in VOOs, only around 2% of these is transferred from the olive fruit to the VOO during the mechanical extraction process, while the other 98% is retained in the olive cake and in the vegetation water. Therefore, the final phenol concentration in VOO may be rather low, ranging from 20 to a maximum of 800 mg kg^-1^ [15]. As the consequence, the intake of phenolic compounds from olive oil is rather low for humans, compared to that from other phenolic sources [16]. Indeed, increasing phenolic content in VOO and EVOO by using innovative cropping techniques and processing methods becomes relevant.

One possible way to reach the above objective is by using phenolic extracts in the form of additives. Some authors [15] added different concentrations of individual and combined phenolic compounds to VOO lipid matrix of a refined olive oil, while other authors [17] studied the enrichment of edible oils with a phenolic extract obtained from olive leaves. Bouaziz et al. obtained similar results by adding olive leaf extracts to husk olive oils [18]. Suarez et al. evaluated some possible strategies for the development of phenol-enriched VOO, by using phenolic compounds obtained from olive cake [19].

Another possible strategy would be to exploit abiotic stresses, which produce an effect on PAL activity and, therefore, on the concentration of phenols within the plant. In this respect, research has shown that it was possible to increase the concentration of phenolic compounds in VOOs by inducing water stresses to olive trees [20; 21].

A third line of action may be to adjust the cropping techniques of olive trees in order to promote a higher content in phenols. D’Amato et al. have studied the possibility of producing Se-enriched EVOO [22], by spraying the olive tree canopy at flowering with sodium selenate (Se-fertilization). The above authors showed that such Se-enrichment produced effects also on oxidative and water stresses, playing a role on the process of plant senescence [23; 24; 25]. Furthermore, other researches have shown that the biofortification with Se may increase PAL synthesis and/or PAL activity in several plant species [26; 27; 28]. As a consequence, it could be expected that Se-fertilization of olive trees may bring to a higher content of phenols in VOO, although no reference is available in this respect.

The aims of this research were to assess the effects of Se-fertilization on yield quality of olive trees (*Olea europea* L. cv. Leccino). In particular, this study considered the content of pigment and total phenols in leaves and drupes, the overall profile of phenolic compounds in olive drupes and the content of phenols in EVOO.

## Materials and methods

### Plant material, site characteristics, and agronomic treatments

This research was carried out in Central Italy (Torgiano, 220 m a.s.l., 43°02′ N and 12°43' E) in 2013 and 2014. Climatic conditions during the two years were monitored by the meteorological station at the Experimental Farm of the Department of Agricultural, Food and Environmental Sciences (University of Perugia), which is located nearby. The soil, derived from calcareous marl and classified as Typic Haploxerept (Soil Survey), is characterised by an alkaline pH (8.1), a loamy texture (sand 41%, silt 34%, clay 25%), 10.0 g kg^-1^ organic C, 2.0 g kg^-1^ total N, 5.5 mg kg^-1^ available P, and 190 mg kg^-1^ exchangeable K. Soil total Se concentration was 0.01 mg kg^-1^.

The seventeen years-old olive trees (cv. Leccino), were trained on monocone training system (about 4 m high) and with planting distances of 6.0 m between the rows and 3 m along the rows (North-South). The trees, pruned annually in the spring, were fertilized with manure. Irrigation was carried out in the morning, from late July to mid-September, using drip lines (two drippers per tree, each through a flow rate of 4 L h^-1^) for a total amount of 110 m^3^ ha^-1^; irrigation interventions were implemented according to measurements of the leaf water potential made by pressure chambers (Scholander chamber).

### Treatment with selenium

At the end of April (26^th^ of April 2013 and 28^th^ April 2014), before flowering, 21 randomly selected trees in 2013 and 21 randomly selected trees in 2014, were sprayed with a solution containing 100 mg L^-1^ of Se. This solution was obtained by dissolving sodium selenate (Sigma-Aldrich, Milan, Italy) in water. For each treatment, 0.5% of the wetting agent “Bagnante” (Albamilagro International S.p.A., Parabiago, MI, Italy) was added. Each plant was treated with 10 L Se solution and wrapped with filter paper to prevent the solution from dripping onto the soil. The paper was weighed before and after spraying to calculate the amount of solution that was absorbed by the plant, which was indeed 7.3 ± 1.4 L. On the other hand, twenty randomly selected ‘control’ trees were sprayed with the same technique, but with a water solution containing only the wetting agent.

### Olive harvesting

Olive drupes and leaves were hand-sampled at 15 days interval, starting from mid July to early November. Sampling dates for the two years are reported in Table 1.

**Table 1:** Sampling dates for the two experimental years. The last sampling date was at crop harvest in both years (in brackets the days after full bloom).

Code	Sampling date	
	2013	2014
1	15^th^ Jul (53)	18^th^ Jul (58)
2	1^nd^ Aug (70)	4^th^ Aug (76)
3	19^th^ Aug (88)	20^th^ Aug (92)
4	2^nd^ Sep (102)	4^th^ Sep (107)
5	18^th^ Sep (118)	15^th^ Sep (118)
6	3^rd^ Oct (133)	2^th^ Oct (135)
7	17^th^ Oct (47)	20^th^ Oct (153)
8	4^th^ Nov (165)	3^rd^ Nov (167)

The olive oils were extracted from olives the day after the last harvest (beginning of November). The oil samples were stored in closed, dark glass bottles in a refrigerator at 4°C.

### Water content of fruits

Samples of approximately 50 g for fresh fruits were dried in a hot air circulation oven at 105°C for 12–18 hours, to constant weight.

### Virgin olive oil mechanical extraction process

The experiments were conducted with an industrial plant using a TEM 200 system (Toscana Enologica Mori, Tavarnelle Val di Pesa, Florence, Italy) composed of a hammer mill, a malaxer with a gas controller system (with a working capacity of 200 kg of olives), and a two-phase decanter; for the separation of olive oil, a UVPX 305 AGT 14 centrifuge (Alfa Laval S.p.A., Tavarnelle Val di Pesa, Florence, Italy) was employed. The extraction was performed on a sample of 150 kg of olives and the malaxation was carried out by using a top-covered malaxing machine, for 30 min at 25°C, which is commonly found in industrial plants.

### Extraction of Phenylalanine Ammonia-Lyase (PAL) in fruits

Samples of olive pulp (from 30 olives) were ground to a paste and used for biochemical assay. The pastes (1.0 g) were homogenized for 30 s in a cold 0.05 M potassium phosphate buffer (25 mL; pH 6.6) with 0.25 g of Triton X-100 using an Ultra-Turrax High-Speed Homogenizer (IKA Labortechnik, Staufen, Germany). Polyvinylpolypyrrolidone (PVPP), 25 mg, was added and the suspension was centrifuged at 4°C for 15 min at 9,000 rpm. The supernatant, stored on ice, was filtered through glass wool, placed on ice, and used as the source of crude enzyme.

### Assay of PAL activity

PAL activity in the enzyme extract was measured using the method of Lister et al. (1996) [29]. The reaction mixture comprised 1 mL of enzyme extract, 1 mL of 50 mM phenylalanine, and 1 mL of 150 mM Tris buffer (pH 8.4) and incubated at 37°C in a water bath for 1 h. PAL enzyme activity was assessed by measuring absorbance at 290 nm using a UV spectrophotometer (Varian Cary 210, Varian, Walnut Creek, CA, USA). Triplicate assays were performed for each enzyme extract. PAL enzymatic activity was expressed in μM cinnamic acid liberated per mg of protein per hour (μmol cinnamic acid ((mg of protein h)^-1^). Protein content in the enzyme extracts was measured using the method Bradford, using bovine serum albumin (BSA) as a standard [30].

### Determination of total selenium content in fruits and oils

The determination of total selenium content in fruits was made on samples dried in a hot air circulation oven at 65°C, for approximately 8–10 hours to constant weight. The fruits were milled into paste, while the analysis of oil was conducted on unaltered samples. Acid digestion of paste and oil samples (0.25 g each) was performed with a mixture of 10 mL of HNO_3_ and H_2_O_2_ (8:2 v/v) that were added to each vessel and the heating for 30 min to 200°C was applied [31]. All steps were performed at an applied power of 1000 W. Maximum pressure was set at 120 bar. Fruits and Oils Se concentrations were determined with graphite furnace atomic absorption spectrophotometry using a Shimadzu AA-6800 apparatus (GF-AAS; GFA-EX7, Shimadzu Corp., Tokyo, Japan) with deuterium lamp background correction and a matrix modifier (Pd(NO_3_)_2_, 0.5 mol L^−1^ in HNO_3_). All analyses were carried out in triplicate.

### Determination of total poliphenols in olive fruits

The methodology to extract phenols followed an adaptation of the method of Fantozzi and Montedoro, 1978 [32]. Analyses were performed in triplicate. Approximately, 1 g of ground olive pulp from 25 olives was mixed in duplicate with 40 mL of hexane and the mixture was homogenised using an Ultra-Turrax homogeniser for 2 min; the upper phase was recovered and the extraction was repeated twice successively with the lower phase to allow removal of pigments and most of the lipids. Phenolic compounds were extracted twice with 80 mL of a mixture of methanol/water 80%/20% (v/v) containing 400 ppm of sodium metabisulphite. The aqueous methanol phases were combined and filtered. The supernatants containing the phenolic compounds were filtered through 0.45 μm syringe filters.

Aliquots of these extracts were assayed for total phenol content using the Folin-Ciocalteu reagent according to the Folin–Ciocalteu method [33]. The extract (100 μL) was mixed with 50 μL of Folin–Ciocalteu reagent. After mixing thoroughly, 300 μL of saturated sodium carbonate solution was added and the mixture was shaken for 0.5 min. Finally, the solution was brought up to a volume of 1 mL with distilled water. After 30 min of reaction at ambient temperature in dark, the absorbance was measured at 765 nm in a UV–Vis spectrophotometer (Varian Cary 210). Based on the standard curve prepared with gallic acid, the amount of total phenolic compounds was calculated and estimated as mg gallic acid kg^-1^ FW of olive pulp [34].

### Determination of chlorophylls and carotenoids in fruits

Pigments (chlorophylls and total carotenoids) were also determined in fruits. Two grams of fruit samples (exocarp and mesocarp) were dissolved in 25 mL of 80% acetone in water. The solution was filtered through a double layer of cheese cloths and absorbances at 663.2, 646.5, and 470 nm were determined using a Varian Cary 210 spectrophotometer. Analyses were performed in triplicate. The concentrations of pigments were calculated according to the equations of Lichtenthaler and Wellburn [35] as follows:

$$C_{a}(mg\;L^{-1})=(12.25\times A_{663.2})-(2.79\times A_{646.8})$$

$$C_{b}(mg\;L^{-1})=(21.5\times A_{646.8})-(5.1\times A_{663.2})$$

$$C_{x+c}(mg\;L^{-1})=(1000\times A_{470}-1.82\times C_{a}-85.02\times C_{b})/198$$

where: C_a_ = Clorophyll a, C_b_ = Clorophyll b, C_x+b_ = Total carotenoids

### Phenolic compounds in VOO

The analysis of VOO phenols was conducted by LLE as reported in Antonini et al. [36] with the exception that the quantitative analysis of the phenolic compounds was conducted using only DAD. Briefly, 22.5 g of VOO were mixed with 10 mL of a solution of methanol/water (80/20 v/v) and homogenized in an Ultra-Turrax T 25 homogenizer (IKA Labortechnik, Staufen, Germany) at 17,000 rpm for 2 min, then after centrifugation at 3,000 rpm for 10 min the supernatant was recovered. The operations were repeated twice, after the two supernatants were combined and concentrated to Rotavapor at 37°C until syrupy consistency. The extract was taken with methanol and the solvent was evaporated to dryness using a nitrogen stream. The extract analysis, after solubilization with 1 mL methanol and filtration over a 0.22 μm PVDF syringe filter, was performed by reversed-phase HPLC. The HPLC analysis was performed using an Agilent Technologies system Mod. 1100, consisting of a vacuum degasser, a quaternary pump, an autosampler, a thermostated column compartment, a DAD, and a fluorescence detector (FLD), controlled by ChemStation (Agilent Technologies, Palo Alto, CA. USA), also used to process the chromatographic data. A C_18_ column Spherisorb ODS-1 250 mm × 4.6 mm with a particle size of 5 μm (Waters, Milford, MA, USA) thermostatted at 25°C was used to separate the phenolic compounds. The mobile phase consisted of 0.2% acetic acid (pH 3.1) in water (solvent A)/ methanol (solvent B), and the gradient changed as follows: 95% A for 2 min, 75% A in 8 min, 60% A in 10 min, 50% A in 16 min, 0% A in 14 min, and maintained for 10 min, then return to the initial condition and equilibration in 13 min; the total running time of the HPLC analysis was 73 min. The flow rate was 1 mL/min. The phenolic compounds were quantitatively determined with DAD at 278 nm; FLD, operated at an excitation wavelength of 280 nm and an emission of 339 nm, was employed only to confirm the lignans peaks.

### Data analysis

Results for all experimental variables were submitted to three-way repeated measures ANOVA, by considering the 'treatment', 'sampling date' and 'year' as experimental factors. The 'tree' was included as the 'subject' random effect to account for the repeated measures in each tree and the variance was assumed as proportional to the mean, to account for heteroscedasticity. The analyses were performed by using the lme() function in R [37]. In order to summarise the results obtained in olives, the two-way matrix relating to Se content, PAL activity, total phenol content, total chlorophyll content, and carotenoid content for all observations (i.e. the data for all combinations 'treatment x sampling date x year') was submitted to Principal Component Analysis (PCA). Data were standardised previous to the analysis and results were displayed on a distance-biplot [38].

## Results and discussion

In the year 2013 the total amounts of olives harvested were 527 kg and 519 kg for the control and Se-treated trees, respectively, while in the 2014 were 469 kg and 474 kg for the control and Se-treated trees, respectively.

Concerning weather conditions, the two experimental years showed contrasting characteristics. The year 2013 was characterised by a low rainfall level in summer (581 mm from April to October, with the lowest value, 27.2 mm, in August), while 2014 was more rainy, with rather abundant rainfall levels in summer (614 mm from April to October, with the lowest value, 44.6 mm, in August). Rainfall level in June-August was 56 mm in 2013 and 211 mm in 2014. The average temperature was 19.5 in 2013 and 18.9°C in 2014. It is likely that, in spite of the irrigation, the level of water stress for olive trees, as measured by the relative water content, was higher in 2013 than in 2014.

Considering olive drupes, total Se-content, PAL activity and total chlorophylls were significantly affected by the interaction ‘Se-fertilization × year × sampling date' (P levels respectively equal to 0.0001, 0.0052 and 0.0001). Otherwise, the content in carotenoids was significantly affected by the interaction ‘Se-fertilisation × sampling date' (P = 0.0123), while the content in phenols was significantly affected only by ‘Se-fertilisation’ (P = 0.0001) and such an effect was consistent across years and sampling dates (the interactions ‘Se-fertilisation × sampling date’ ‘Se-fertilisation × year’ and ‘Se-fertilisation × sampling date × year’ were not significant).

**Fig 1:**
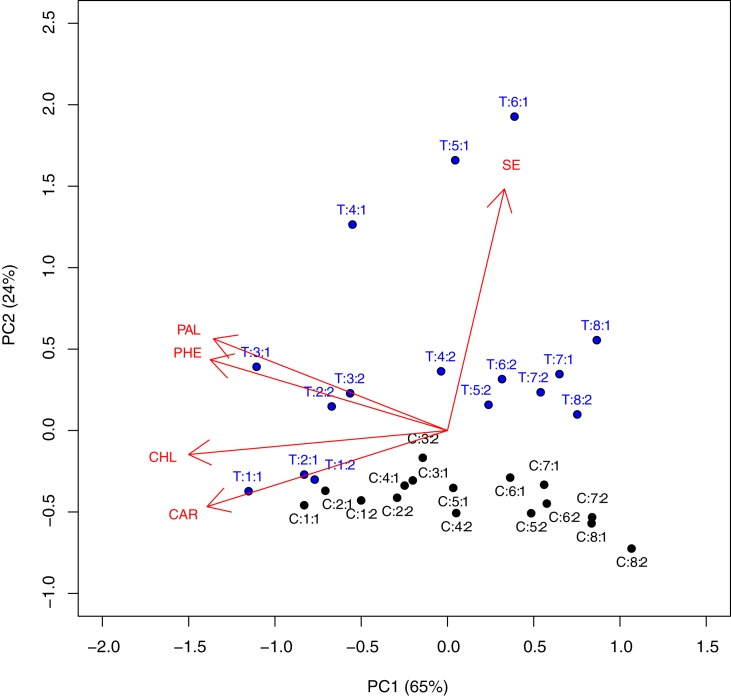
Principal component analysis for the content of Se (SE), PAL activity (PAL), total phenols (PHE), total chlorophylls (CHL), and carotenoids (CAR) in drupes harvested from olive trees, following a fertilization with sodium selenate before flowering. Symbols represent the combinations of treatment (T = treated, C = untreated): sampling date (from 1 to 8, for details see Table 1): year (1 = 2013 and 2 = 2014).

The whole set of results relating to olive drupes was summarized by using a PCA, which explained 89% of the total variance of data with two principal components (65% and 24%, respectively). The resulting biplot shows that the concentration of Se is mainly related to the positioning of observations along the second component (y-axis) (Fig 1).

In this respect, treated trees were consistently located at the top and the untreated trees at the bottom in Fig 1, although such a difference was small at 1^st^ and 2^nd^ sampling date, while it became very high at 4^th^, 5^th^, and 6^th^ sampling dates and decreased afterwards, while approaching the date of final harvest (Table 2). The average concentration in Se in the two years was 12.2 times higher in Se-fertilised trees, with respect to untreated trees, and such a difference was particularly high at the 4th, 5th, and 6th sampling dates in 2013.

**Table 2:** Changes in se-concentration in *Olea europea* L. olive fruits (ng Se g^-1^ DW).

	2013				2014			
Code	Untreated	SD	Se-enriched	SD	Untreated	SD	Se-enriched	SD
1	10.3	1.5	15.7 *	3.2	13.4	4.8	28.8 *	3.2
2	11.3	0.9	65.3 *	2.8	13.6	1.7	43.9 *	4.4
3	16.0	4.4	58.7 *	1.6	19.0	3.1	52.6 *	3.0
4	15.3	3.5	347.3 *	50.4	16.6	1.8	135.6 *	16.5
5	18.0	5.6	590.3 *	144.7	18.4	4.6	116.1 *	8.1
6	15.7	4.7	696.7 *	144.7	17.0	3.5	190.9 *	4.2
7	18.3	3.5	232.0 *	33.2	18.3	3.0	191.8 *	5.7
8	18.0	3.0	330.3 *	18.8	18.5	2.5	178.7 *	22.2

SD: standard deviations of three determinations. Data followed by the asterisks are significantly different from the untreated control at the same year and sampling date (P < 0.05).

As expected, PAL activity and total phenols are highly correlated (vectors show a very low angle between each other in Fig 1) and they are mainly related to the positioning of observations along the first component (x-axis), although both vectors show a positive component along the y-axis. Indeed, both PAL activity and phenol concentration tended to decrease with sampling dates, although they were generally higher in Se-fertilized trees, with respect to the untreated controls (see Tables 3 and 4). In the case of phenols, the increase relating to Se-fertilization was rather consistent (no interactions between 'treatment' and other experimental factors were significant) and it was, on average, equal to +17.6%, with up to +27.4% in olive fruits of the 5^th^ sampling date in 2014.

**Table 3:** Changes in PAL activity (L-Phenylalanine Ammonia-Lyase) expressed as μM cinnamic acid (mg of protein × h)^-1^, of *Olea europea* L. olive fruit.

	2013				2014			
Code	Untreated	SD	Se-enriched	SD	Untreated	SD	Se-enriched	SD
1	118.00	7.5	136.14	9.6	138.74	6.1	145.38	0.8
2	122.26	31.7	144.29	9.6	127.69	6.9	160.43 *	5.4
3	113.17	28.2	180.96 *	10.7	135.13	3.7	154.77 *	18.3
4	125.95	54.5	167.19 *	32.3	92.58	5.5	119.87	1.4
5	101.80	4.9	137.72 *	15.8	84.22	4.4	103.91	3.8
6	108.09	4.6	116.27	17.3	80.39	1.5	97.52	1.9
7	101.84	7.9	101.27	3.4	66.43	8.1	89.60	5.7
8	81.80	5.9	92.60	5.7	53.68	2.3	90.92 *	0.6

SD: standard deviations of three determinations. Data followed by the asterisks are significantly different from the untreated control at the same year and sampling date (P < 0.05).

**Table 4:** Changes in total phenols (mg kg ^-1^of gallic acid FW) of *Olea europea* L. olive fruits.

	2013				2014			
Code	Untreated	SD	Se-enriched	SD	Untreated	SD	Se-enriched	SD
1	19587	306	20709	718	14946	611	17100	679
2	18456	535	22456	1200	15159	494	19292	737
3	17411	515	21856 *	530	15841	611	19854 *	156
4	16291	703	20604 *	829	15482	1309	18905 *	979
5	16645	893	16629	916	13253	614	16890 *	112
6	13805	1773	15893	433	13795	1094	16490	985
7	12577	1002	12781	1367	12298	1210	14910	867
8	10181	627	11330	935	9719	1509	11781	288

SD: standard deviations of three determinations. Data followed by the asterisks are significantly different from the untreated control at the same year and sampling date (P < 0.05).

The contents in chlorophyll (a+b) and carotenoids are highly correlated between each other (small angles in Fig 1) and, similarly to phenols and PAL, they are located to the left side of the x-axis, although they present a negative component along the y-axis (Fig 1). Therefore, the contents in pigments tended to decrease with sampling date (see Table 5).

**Table 5:** Changes in pigments (mg g^-1^ FW) of *Olea europea* L. olive fruits in the years 2013 and 2014.

	**2013**							
	Chlorophyll a+b				Carotenoids			
Code	Untreated	SD	Se-enriched	SD	Untreated	SD	Se-enriched	SD
1	0.21	0.01	0.24 *	0.02	0.033	0.007	0.037	0.006
2	0.23	0.02	0.26 *	0.01	0.027	0.001	0.030	0.005
3	0.12	0.01	0.22 *	0.02	0.023	0.002	0.024	0.005
4	0.10	0.01	0.14 *	0.01	0.027	0.005	0.019 *	0.006
5	0.09	0.01	0.09	0.01	0.022	0.003	0.019	0.006
6	0.07	0.01	0.08	0.01	0.014	0.001	0.013	0.001
7	0.05	0.01	0.04	0.01	0.013	0.001	0.012	0.001
8	0.04	0.01	0.04	0.01	0.014	0.001	0.010	0.001
	**2014**							
	Chlorophyll a+b				Carotenoids			
Code	Untreated	SD	Se-enriched	SD	Untreated	SD	Se-enriched	SD
1	0.18	0.02	0.20 *	0.01	0.029	0.001	0.031	0.002
2	0.14	0.02	0.16 *	0.01	0.027	0.003	0.023	0.002
3	0.11	0.01	0.15 *	0.01	0.021	0.005	0.021	0.001
4	0.12	0.01	0.10	0.01	0.023	0.002	0.015 *	0.002
5	0.07	0.01	0.09 *	0.01	0.017	0.002	0.013	0.002
6	0.05	0.01	0.08 *	0.01	0.015	0.001	0.013	0.001
7	0.04	0.01	0.06 *	0.01	0.012	0.001	0.012	0.001
8	0.04	0.02	0.06	0.01	0.011	0.001	0.012	0.001

SD: standard deviations of three determinations. Data followed by the asterisks are significantly different from the untreated control at the same year and sampling date (P < 0.05).

It is confirmed that VOOs obtained from Se-fertilised trees showed higher contents in phenols with respect to untreated controls. In more detail, the increase in total polyphenols was 44% and 38% in 2013 and 2014, respectively (Table 6).

**Table 6:** Content of phenolic compounds (mg kg^-1^ of oil) in the VOOs from Leccino cultivar treated and untreated with Se and harvested at olive maturation in the year 2013 and 2014.

	**2013**			
	Untreated	SD	Se-enriched	SD
3.4-DHPEA	1.80	0.03	1.71 *	0.03
*p*-HPEA	1.95	0.03	1.38 *	0.02
3.4-DHPEA-EDA	185.6	3.2	298.6 *	4.1
*p*-HPEA-EDA	16.7	0.2	21.2 *	0.3
(+)-1-Acetoxypinoresinol	3.47	0.02	3.09 *	0.04
(+)-Pinoresinol	8.1	0.1	8.15	0.06
3.4-DHPEA-EA	48.0	0.5	48.7	0.7a
Ligstroside aglycon	3.08	0.06	4.74 *	0.08
**Total phenols**	**268.7**	**3.3**	**387.5** *	**4.2**
	**2014**			
	Untreated	SD	Se-enriched	SD
3.4-DHPEA	1.94	0.02	1.59 *	0.01
*p*-HPEA	2.59	0.02	2.31 *	0.07
3.4-DHPEA-EDA	152.6	0.5	233.5 *	2.9
p-HPEA-EDA	41.2	0.1	49.2 *	1.0
(+)-1-Acetoxypinoresinol	25.5	0.1	27.1 *	0.1
(+)-Pinoresinol	9.4	0.2	11.07 *	0.04
3.4-DHPEA-EA	52.4	0.3	69.1 *	0.9
Ligstroside aglycon	4.7	0.1	7.0 *	0.4
**Total phenols**	**290.4**	**0.6**	**400.8** *	**3.2**

SD: standard deviations of three determinations. Data followed by the asterisks are significantly different from the untreated control at the same year and sampling date (P < 0.05).

However, such a total increase in phenols should be better characterised by considering the different chemical classes (Tab. 6). In both years, phenolic alcohols slightly decreased, while secoiridoids derivatives increased. Considering the profile in terms of percentage variations, it is very interesting to note that the phenolic substances that were most positively affected by the treatment were 3.4-DHPEA-EDA (dialdehydic form of decarboxymethyl elenolic acid linked to hydroxytyrosol or oleacein), which was increased on average by 57% (61% and 53% for the first and the second year, respectively) and ligustroside aglycone, with an average increment of 51% (54% in 2013 and 49% in 2014) (Table 6).

The *p*-HPEA-EDA (dialdehydic form of decarboxymethyl elenolic acid linked to tyrosol or oleocanthal), another important secoiridoid derivative, showed a lower increase (27 and 19%, respectively). The 3,4-DHPEA-EA was unchanged in the first year, while it increased considerably (32%) in the second year. Lignans did not show any change relating to Se-treatment. These results are relevant, because several studies show that 3.4-DHPEA-EDA is the main phenolic compound in EVOOs and it is characterised by a high antioxidant activity, which contributes to a good shelf-life and hypothensive and anti-inflammatory effects on human health [39; 40].

The average content of Se in EVOOs from Se-fertilised plants was 120 μg kg^-1^; considering a mean daily consumption of 20 g of EVOO and an Acceptable Daily Intake (ADI) of 55–70 μg Se day^-1^, the actual daily intake of Se would be 2.4 μg, corresponding to 4.3% and 3.4% of the ADI, respectively (Se), which seems to be a safe amount [41; 42].

In conclusion the practice of Se-fertilisation in olive trees (cv. Leccino) grown in years with different climatic trends positively influenced the phenolic composition of extra virgin olive oil, both in terms of total phenolic content and above all, in terms of 3,4-DHPEA-EDA. Therefore, this agronomic practice could represent a valid tool for improving the chemical profile of poor cultivars in terms of polyphenols, and, in particular, in terms of oleuropein derivatives. Furthermore, such an effect was consistent in two experimental seasons with contrasting weather patterns, particularly in terms of different water availability. Indeed, the effect of Se-fertilisation helped maintaining a good phenolic profile, particularly in the year with more abundant rainfall level, while it is well known that phenolic accumulation in drupes is higher in years with warm temperatures and low water availability [43]

It is relevant to note that the improvement of phenolic profile in EVOOs was obtained by treating olive trees, with no direct enrichment of the final commercial product, which would be prohibited for EVOOs, considering that only physical treatment methods are allowed during the mechanical extraction process. It is also important to highlight that this procedure may be particularly useful with EVOOs characterised by a poor phenolic profile, which cannot meet the European Food Safety Authority (EFSA) statement about the admissibility of the health claim for EVOOs. Indeed, a well planned Se-fertilisation before flowering may help these EVOOs reach the minimum content of 5 mg of hydroxytyrosol and its derivatives (e.g. the oleuropein complex and tyrosol) in 20 g of product (corresponding to 250 mg kg^-1^ of oil) (EU, 2012) [39].

Future work should confirm that the above results can be reached also in other olive cultivars, apart from Leccino.

## Supporting information

S1 Table Original dataset. Table of original data.
(XLSX)

## Abbreviations

VOO: virgin olive oil
EVOO: extra-virgin olive oil
ANOVA: analysis of variance
PAL: L-Phenylalanine Ammonia-Lyase
ANOVA: analysis of variance
PCA: Principal component analysis
3.4-DHPEA-EDA: dialdehydic form of decarboxymethyl elenolic acid linked to hydroxytyrosol
*p*-HPEA-EDA: dialdehydic form of decarboxymethyl elenolic acid linked to tyrosol
